# Solutions to the Cocktail Party Problem in Insects: Selective Filters, Spatial Release from Masking and Gain Control in Tropical Crickets

**DOI:** 10.1371/journal.pone.0028593

**Published:** 2011-12-06

**Authors:** Arne K. D. Schmidt, Heiner Römer

**Affiliations:** Department of Zoology, Karl-Franzens-University, Graz, Styria, Austria; Lund University, Sweden

## Abstract

**Background:**

Insects often communicate by sound in mixed species choruses; like humans and many vertebrates in crowded social environments they thus have to solve cocktail-party-like problems in order to ensure successful communication with conspecifics. This is even more a problem in species-rich environments like tropical rainforests, where background noise levels of up to 60 dB SPL have been measured.

**Principal Findings:**

Using neurophysiological methods we investigated the effect of natural background noise (masker) on signal detection thresholds in two tropical cricket species *Paroecanthus podagrosus* and *Diatrypa* sp., both in the laboratory and outdoors. We identified three ‘bottom-up’ mechanisms which contribute to an excellent neuronal representation of conspecific signals despite the masking background. First, the sharply tuned frequency selectivity of the receiver reduces the amount of masking energy around the species-specific calling song frequency. Laboratory experiments yielded an average signal-to-noise ratio (SNR) of −8 dB, when masker and signal were broadcast from the same side. Secondly, displacing the masker by 180° from the signal improved SNRs by further 6 to 9 dB, a phenomenon known as spatial release from masking. Surprisingly, experiments carried out directly in the nocturnal rainforest yielded SNRs of about −23 dB compared with those in the laboratory with the same masker, where SNRs reached only −14.5 and −16 dB in both species. Finally, a neuronal gain control mechanism enhances the contrast between the responses to signals and the masker, by inhibition of neuronal activity in interstimulus intervals.

**Conclusions:**

Thus, conventional speaker playbacks in the lab apparently do not properly reconstruct the masking noise situation in a spatially realistic manner, since under real world conditions multiple sound sources are spatially distributed in space. Our results also indicate that without knowledge of the receiver properties and the spatial release mechanisms the detrimental effect of noise may be strongly overestimated.

## Introduction

Acoustic communication and hearing in humans and non-human animals did not evolve in sound-proof rooms, but under real-world conditions which are often characterized by a considerable amount of noise, and the information to be transmitted between signaler and receiver(s) can be profoundly constrained. Such noise is either caused by non-biological sources such as wind, running water etc., or by heterospecific signalers where the sum of all emitted sound signals produces an acoustic background in which the conspecific signal has to be detected and discriminated from irrelevant sound [1], [2], [3], [4]. However, at the ear of a receiver the sound waves of all relevant and irrelevant signals are mixed, and subsequently have to be segregated by the auditory system into individual sound sources. The well-known cocktail party problem [5], [6] describes the difficulty of human listeners to perceive speech under noisy (social) conditions. How humans solve the fundamental problem of segregating the different sound sources has a long history of research [7], [8].

Comparable studies on animals in different taxa have shown that they have to solve rather similar problems, in particular those that live in larger aggregations and social groups [9], [10], [11], (see Bee and Micheyl for an excellent review dealing with cocktail party-like problems in animal communication [12]). There are several solutions on hand to improve signal detection and/or discrimination under background noise which can be divided into those related to either the signaler or receiver. Signalers could engage in acoustic niche partitioning in time and space [13], [14], [15], shift their song frequency into a less disturbed range [16], [17], [18], increase the amplitude of their signal (the so-called Lombard-effect [19]), use multimodal or alternative signals [20], or increase signal redundancy and duration [21], [22], [23], [24] to counteract the masking of their signal by noise. Receivers may change the characteristics of their peripheral or central auditory filters [25], [26], [27], [28], or even change the best frequency of filters depending on masking noise conditions [29]. They could also use automatic gain control mechanisms to increase the contrast between signal and background [30], [3].

One further mechanism, referred to as spatial release from masking [9], can usually improve the detection and discrimination of signals in noise when the masker is spatially separated to some degree from the signal. This mechanism is based on the directionality of the receiveŕs hearing system and contributes to sound source segregation. Numerous studies have demonstrated that this mechanism can improve speech perception in human listeners [6], [31], [32]. Similarly, spatial release from masking improves the detection and discrimination of conspecific signals from heterospecifics in anurans [33]; for further studies on spatial unmasking in signal detection tasks in vertebrates see Bee and Micheyl 2008 [12].

However, surprisingly little is known for insects on this mechanism. Ronacher and Hoffmann [34] investigated the influence of amplitude modulated noise on the recognition of species-specific communication signals in a grasshopper behaviourally, and found little evidence for spatial release from masking. They explained their negative finding with the particular mode of processing signals for pattern recognition in grasshoppers (summation of signals from both auditory sides; [35]). However, this is not the case in crickets and katydids [36], [37], [38], [3], and although spatial release from masking was not addressed directly in these studies, they nevertheless indicate that the mechanism may work effectively in these taxa.

Spatial release from masking experiments are usually performed in the laboratory (either behaviourally or physiologically) by determining masked detection and/or discrimination thresholds, when signal and masker are co-located and afterwards when the masker was spatially separated from the signal. However, these conventional lab experiments do not reflect the real-world listening conditions that many animals face in a chorus, where a receiver is confronted with several masking sources from multiple directions, so that both the masking and unmasking situation differs from the usual experimental setup in the lab.

The aim of the present study was therefore to examine the outcome of spatial release experiments in the lab with the masking condition in the natural habitat of the receiver. We did this by using two cricket species which communicate acoustically in the nocturnal tropical rainforest for which high masking noise levels have been reported [4], [39]. We take advantage of the fact that for acoustic insects experimental approaches are available to examine single neurons of the afferent auditory pathway in a portable preparation, which can be placed at any position outdoors, and its responses to conspecific stimuli under natural background be compared [40], [41], [42]. Our results show that three ‘bottom-up’ mechanisms exist in the afferent auditory pathway of tropical crickets, namely selective frequency filtering, spatial release from masking, and a gain control, which all contribute to the excellent performance of signal detection in high background noise levels. Whereas the conventional masking and spatial unmasking approach in the lab may accurately estimate the maximal benefit that might be produced, they nevertheless strongly overestimate the amount of masking for a receiver under natural settings.

## Materials and Methods

### Ethics statement

The experiments reported in this paper comply with the current animal protection law in Panama. According to these laws, studies on insects do not require approval by a review board institution or ethics committee (Institutional Animal Care and Use Committee Protocol). No specific permits were required for the described field studies.

### Study site and animals

Experiments were carried out in May/June 2010 and February/March 2011 on Barro Colorado Island (9° 9′N, 79° 51′W, Republic of Panama), a 1600-ha forested island within Lake Gatun. Adult male and female crickets of the species *Paroecanthus podagrosus* and *Diatrypa* sp. (Orthoptera: Grylloidea: Eneopterinae) were collected at lights near the research station, kept in a plastic terrarium and fed *ad libitum* on a diet of fish flakes, oats, fruits, lettuce and water.

Most of the experiments and corresponding results have not been carried out with both tropical cricket species to the same extend. Spatial release from masking and gain control experiments were predominantly completed in 2010 with *Paroecanthus podagrosus* only. Outdoor experiments were performed in 2011 where both species were present, albeit the number of individuals of *Diatrypa* sp. (N = 6) was limited.

### Neurophysiology

We conducted neurophysiological experiments, both in the laboratory and outdoors in the tropical rainforest, to investigate the effect of background noise on signal detection in the auditory pathway of tropical crickets. We performed extracellular recordings of the action potential activity of a prominent auditory interneuron (AN1), known for its property of encoding behaviorally relevant information about the male calling song, and its essential role for positive phonotaxis [43]. The experimental approach for these recordings has been described in detail elsewhere [44], [45]. In short, the cervical connectives were exposed and its neuronal activity recorded in a preparation ventral side up, using electrolytically sharpened tungsten hook-electrodes. Neuronal signals were amplified using a custom-made amplifier (Topview Electronic, Weiz, Austria) and digitized at a sampling rate of 40 kHz (PowerLab 4/25, ADInstruments, Sydney, Australia) for offline analysis.

Laboratory experiments were carried out in an acoustically isolated Faraday-cage at ambient temperatures between 24 and 25°C. The background noise level at the preparation was below 28 dB SPL in a frequency range from 2 – 10 kHz.

### Acoustic stimuli

**Figure 1:**
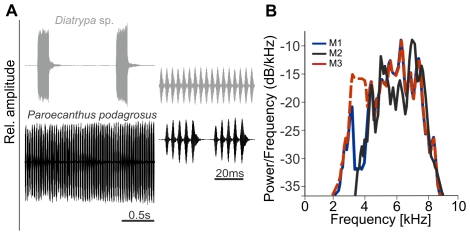
Conspecific stimuli and masker used for experiments. (A) Oscillograms of calling songs of *Diatrypa* sp. and *Paroecanthus podagrosus* at two time scales. (B) Power spectral density of typical background noise recordings (M1, blue and M2, black) of the nighttime rainforest on BCI, Panama. An additional frequency band was digitally mixed with the M1 recording to account for low acoustic energy at frequencies between 3.4 and 4 kHz (M3, red dashed line). Recordings with these spectra were used as maskers in neurophysiological experiments.

The natural calling song of *P. podagrosus* consists of a repetitive series of chirps, build up of 4–6 pulses (pulse duration 3 ms, inter-pulse interval 2 ms) with 14 ms chirp interval; the average pulse rate is 205±17 Hz (Fig. 1A). The total duration of the chirp series is highly variable, lasting from a few seconds up to 2 minutes; the average carrier frequency (CF) is 3.8±0.2 kHz. Similarly, the calling song of *Diatrypa* sp. is a trill composed of a series of pulses (pulse duration 2 ms, inter-pulse interval 1 ms) at a pulse rate of 269±28 Hz; the average CF is 4.0±0.4 kHz.

Synthetic stimuli of the calling songs of both species were computer generated using audio software (CoolEdit Pro 2.0, Syntrillium, Phoenix, USA, now Adobe Audition) with carrier frequencies set to the average best frequency (BF) of receivers sensitivity tuning, which is 3.9 kHz for both species ([28] and this study). Pulse rates were set to 217 Hz (*P. podagrosus)* and 250 Hz (*Diatrypa* sp.*)*; these values are within the range of variation observed in the natural populations. The duration of calling songs for *Paroecanthus podagrosus* was 980 ms, and the inter-stimulus interval (ISI; the time between two songs) 1500 ms. The respective durations for *Diatrypa* sp. were 188 ms and 1100 ms (ISI).

We used prerecorded background noise of the nocturnal rainforest of two different locations with similar frequency distribution as playbacks in masking experiments (masker M1 and M2, Fig. 1B). However, due to only interrupted singing of *Diatrypa* sp. the spectral analysis of these recordings revealed a gap between approximately 3.4 and 4 kHz with reduced acoustic energy, which spans exactly the range of BF in receivers. We therefore digitally filled this gap with an additional noise band of 3.2 to 4.1 kHz (band-pass filtered white noise using CoolEdit; see M3 in Fig. 1B) to reevaluate the performance of the *P. podagrosus* AN1 filter under more challenging masking conditions.

In the laboratory experiments, calling songs and masker were broadcast via an external audio interface (Sound Blaster Extigy, Creative, Jurong East, Singapore), independently attenuated with a step attenuator (Type 837, Kay Elemetrics Corp., NJ, USA) or in case of the masker with a programmable attenuator (PA5, Tucker Davis, Florida, USA) and amplified (stereo power amplifier SA1, Tucker Davis, Florida, USA). Playbacks lasted for 1.2 min of continuous background noise before being repeated in a loop. Masker and conspecific calling songs were broadcast through different speakers (FF1, Tucker Davis; flat frequency response from 1–50 kHz, manufacturer's specification).

### Sensitivity tuning

For *Diatrypa* sp. we determined the receiverś sensitivity tuning in six individuals, using a methodology as described in detail previously for *Paroecanthus podagrosus* [28]. In short, we measured threshold responses of the AN1 using stimuli with carrier frequencies varying from 2 to 6 kHz with a minimum step size of 0.1 kHz.

### Spatial release from masking

The effect of spatial release from masking depends on directional cues of the hearing system. Directionality in crickets is basically provided by a pressure difference receiver, where the anatomical arrangement of the acoustic trachea provides a functional three-input system with sound acting at the tympanum and two tracheal openings (for review see [46]). The peripheral sound entrance via these tracheal openings is mediated by the prothoracic spiracles on both sides of the body. Therefore the opening status of these spiracles was controlled carefully before each neurophysiological experiment and was kept partially open throughout the experiment.

For each individual the unmasked AN1 threshold at 3.9 kHz was determined and signal intensity was set at 20 dB above threshold. In order to determine the SNR at the masked threshold, the masker intensity was subsequently increased in steps of 3 to 1 dB, the neuronal response recorded and stored for offline analysis. AN1 responses were analyzed with Spike 2 software (v5.2, Cambridge Electronic Design, Cambridge, UK). AN1 spikes were detected and separated from other neuronal activity using a custom written spike sorting algorithm [42].

Masked thresholds for the signal in background noise were calculated on the basis of spike rate differences between the stimulus duration and inter-stimulus interval (ISI). We defined a threshold criterion that was reached when the spike rate during the stimulus (i.e. the response due to signal and background noise) first exceeded twice the spike rate during the ISI (i.e. the response of the AN1 due to background noise only). Spike rate calculations were based on an average of 15 stimulus and inter-stimulus repetitions.

Initially, both the signal and masker were broadcast ipsilaterally at 90° off the longitudinal body axis at a distance of 25 cm from the preparation (acoustic free field). After determining the detection threshold with ipsilateral stimulation the masker was moved by 180° to the contralateral side and the experimental protocol was repeated.

### Outdoor experiments

To investigate coding properties of the AN1 and masked thresholds under natural conditions we used the ‘biological microphone’ approach [40], [42] and recorded AP activity of AN1 in preparations placed directly in the nocturnal rainforest. Since the highest acoustic background activity in the frequency band between 2 – 9 kHz was measured between 19:00 and 23:00 h we performed our outdoor experiments within that time window. To compare the results of masked thresholds in the real-world situation with the one under conventional laboratory conditions both sound sources, masker and signal, were broadcast from the same, ipsilateral side, but this time the masker intensity was set to a fixed value of 55 dB SPL (mean background noise level outdoors), and the SPL of the signal was varied using a programmable attenuator (PA5, Tucker Davis).

The aim of the neurophysiological outdoor experiments was to expose the preparation to the natural auditory scene with multiple sound sources from different directions, and to use playbacks with conspecific calling songs to determine SNRs at the masked threshold. The preparation was placed in a small rainforest gap, at a height of about 1 m from the ground. Prior to each experiment, the nocturnal background sound pressure level at the position of the preparation was measured using a sound level meter (NL-21, RION Co., Ltd., Tokyo, Japan) and integrated microphone (UC-52; frequency range 20 Hz to 8 kHz)). These values ranged from 52 to 57 dB SPL in different nights. A speaker (Visaton M10, Haan, Germany) was placed ipsilaterally at 90° off the longitudinal body axis at a distance of 0.5 m to broadcast the conspecific signal at SPLs from 28 to 65 dB SPL.

To correlate the outdoor background noise activity with the bursting pattern of the AN1 neuron, we simultaneously recorded the neural response and the ambient rainforest noise using a condenser microphone (Sennheiser, Hannover, Germany) powered with a Sennheiser MZA 14 and digitized with a PDM670 Marantz recorder (D&M Holdings Inc., Kanagawa, Japan) at a sampling rate of 44.1 kHz.

**Figure 2:**
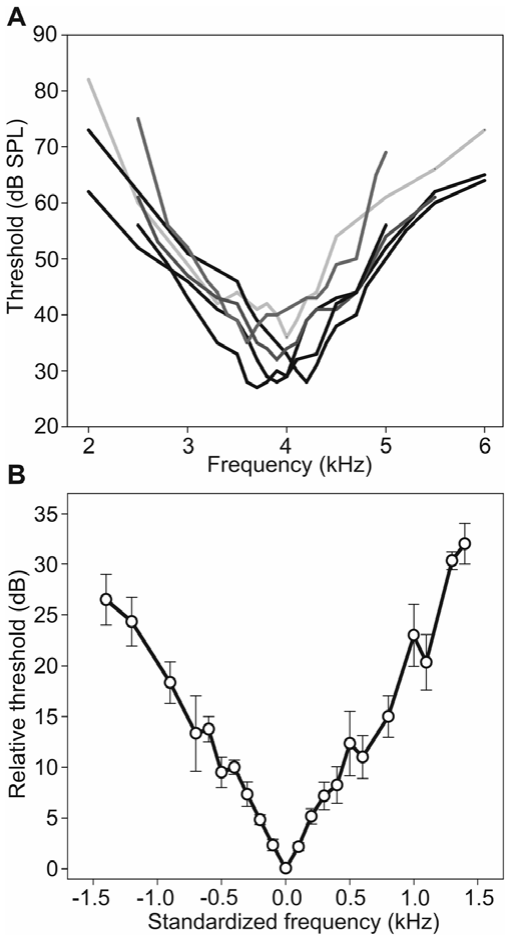
A. Frequency tuning of the AN1 neuron in *Diatrypa* sp. (N = 6). (B) Standardized mean frequency tuning (±SE), with the best frequency of individual tuning curves set at 0 kHz/0 dB and higher thresholds to lower and higher frequencies arranged accordingly.

We hypothesized that elicited action potentials in AN1 should strongly correlate with the auditory filter function of the cricket species [45], [28]. Because the filter is so selective, we expected that very little sound energy in the background noise would be able to reduce this correlation. To test this we took the neural responses of *Diatrypa* sp. caused by rainforest noise and convolved a 30 s lasting spike train of each recording (N = 5) with a Gaussian kernel (σ = 40 ms). We thus obtained a smoothed firing pattern which subsequently was correlated with the RMS amplitude of the corresponding filtered background noise (Matlab, R2008b, The MathWorks Inc., Natick, MA, USA). The used digital filter (FFT size 2048 with Blackman window function) was created with audio software based on the standardized AN1 tuning of *Diatrypa* sp. (Fig.2B). The BF of the filter was set to 3.9 kHz, the species-specific average value of the neuronal frequency filter.

### Gain control

In order to analyze potential effects of the gain control mechanism, i.e. the suppression of AP activity following intense stimulation, we followed the experimental design described for the spatial release from masking experiments. We compared changes of AN1 discharge in background noise-only situations relative to noise intervals between two consecutive calling songs (ISI), both signal and masker broadcast ipsilaterally. This was done for eight individuals of *P. podagrosus* at SNRs of 0, −6, and −9 dB. For each individual at a respective SNR, sections of 6.5 to 23 s (11 s on average) in response to continuous background noise were evaluated followed by calculating spike rates of the corresponding ISI sections (average over 17 trials). Changes in spike rates were expressed in percent, where the activity of the noise-only situation (control) was set to 100%.

Mean values are presented ±SE.

## Results

### Sensitivity tuning

In order to reveal the filter selectivity we determined the frequency tuning of the AN1 neuron in six individuals of *Diatrypa* sp. Individual tuning curves varied with respect to their best frequency (frequency of the lowest threshold) from 3.6 to 4.2 kHz with an average value of 3.9±0.1 kHz (Fig. 2A). The mean sensitivity was 31±1.5 dB SPL, with lowest thresholds varying between 28 and 35 dB SPL.

To reveal the specieś absolute frequency selectivity, we standardized the tuning by defining the BF as 0 kHz and arranging higher thresholds on both sides of the frequency axis relative to this standard (Fig. 2B). Similar to *Paroecanthus podagrosus* [28] the frequency selectivity in *Diatrypa* sp. is characterized by steep symmetric roll-offs to lower (20 dB/−1 kHz) and higher (23 dB/1 kHz) frequencies, respectively. As a quantitative value for the sharpness of frequency tuning we calculated the frequency width 5 dB above threshold at the BF. This value was 450±33 Hz on average.

### Spatial release from masking

Experiments on spatial release from masking were performed with two different background noise recordings as masker of the nocturnal rainforest (M1 and M2; Fig. 1B). However, playbacks of both recordings revealed no differences in the outcome of the results, neither for ipsilateral masked thresholds (t-test, t = −0.921, p = 0.379, N = 5/7) nor for the magnitude of spatial unmasking (Mann-Whitney U test, U = 25.5, p = 0.202, N = 5/7). Thus the results were pooled.

**Figure 3:**
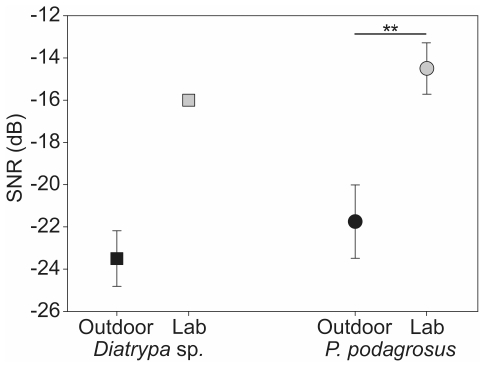
Results of spatial release from masking experiments in the laboratory (*P. podagrosus*). Comparison of SNRs at masked thresholds with masker M1/M2 (black squares; N = 12) and M3 (grey squares; N = 6) for ipsilateral (masker and signal presented from the same side of the recorded AN1) and contralateral masker position (masker spatially separated by 180°).

We determined ipsilateral masked thresholds in 12 male and female *P. podagrosus* individuals. SNRs ranged from −4.5 to −14 dB with a mean of −8.2±0.7 dB (Fig. 3). Subsequently, the masker was spatially separated from the signal (180°) to the contralateral site and the masked threshold was measured again. This improved SNRs on average by 6.1 ±0.6 dB to −14.3±0.9 dB and individual values varied from −9.5 to −20 dB (Fig. 3, Wilcoxon signed-rank test, Z = −3.066, p = <0.001, N = 12).

The masker used in these experiments included relatively little acoustic energy at frequencies between 3.4 and 4 kHz (Fig. 1B), which does explain, in conjunction with the sharply tuned frequency filter, the excellent SNR of the ipsilateral masked threshold. Using the masker M3, where this small frequency range is filled with acoustic energy resulted in a strong decline of the masked ipsilateral threshold by about 8 dB to a SNR of 0.1±0.9 dB (Fig. 3, t-test, t = 7.062, p = <0.001, N = 6), with values ranging from 3 to −3.5 dB.With this masker, the amount of spatial unmasking even increases when the masker was broadcast from contralateral. The mean SNR was −8.8±1.3 dB which lead to an ipsi-contra-difference of 8.7 dB (paired t-test, t = 6.068, p = 0.002, N = 6).

### Outdoor experiments

**Figure 4:**
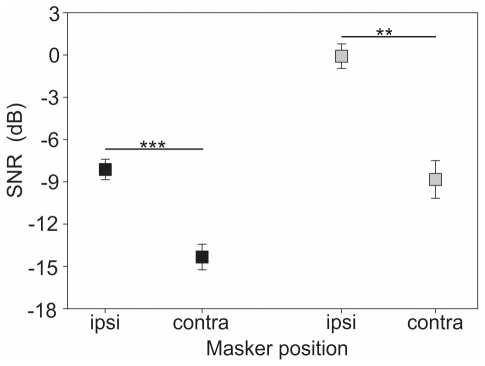
Comparison of SNRs at masked thresholds outdoors and in the laboratory. (*P. podagrosus* lab N = 5, outdoor N = 6*; Diatrypa* sp. lab N = 1, outdoor N = 5). Note the difference in SNRs in the real world situation and laboratory, although the masker M2 recorded at the site where outdoor experiments were performed was very similar spectrally and with respect to average intensity (55 dB SPL).

We complemented the conventional masking experiments in the laboratory with neurophysiological studies in the insectś natural habitat. Measurements of background noise for the 11 experimental nights revealed an average noise level of 55±0.5 dB SPL with variations between different nights ranging from 52 to 57 dB SPL. Surprisingly, masked thresholds of AN1-preparations of *P. podagrosus* and *Diatrypa* sp. in the natural habitat revealed very low SNRs of −21.8±1.7 dB and −23.5±1.3 dB, respectively. These values were much lower than those reported in the laboratory (Fig. 4), where for *P. podagrosus* the masked threshold yielded only values corresponding to SNRs of −14.5±1.2 dB (t-test, t = −3.281, p = 0.01, N = 6/5). Importantly, this difference was not due to differences in hearing thresholds (absolute sensitivity) between preparations used for outdoor and laboratory experiments, which were rather similar with an average of 34 dB SPL and 35 dB SPL, respectively.

For a comparison with *Diatrypa* sp. we have only been able to perform one experiment in the lab, but the masked threshold at a SNR of −16 dB compared with −23.5±1.3 dB for five preparations outdoors is rather similar to the outcome of the experiments in *P. podagrosus*.

**Figure 5:**
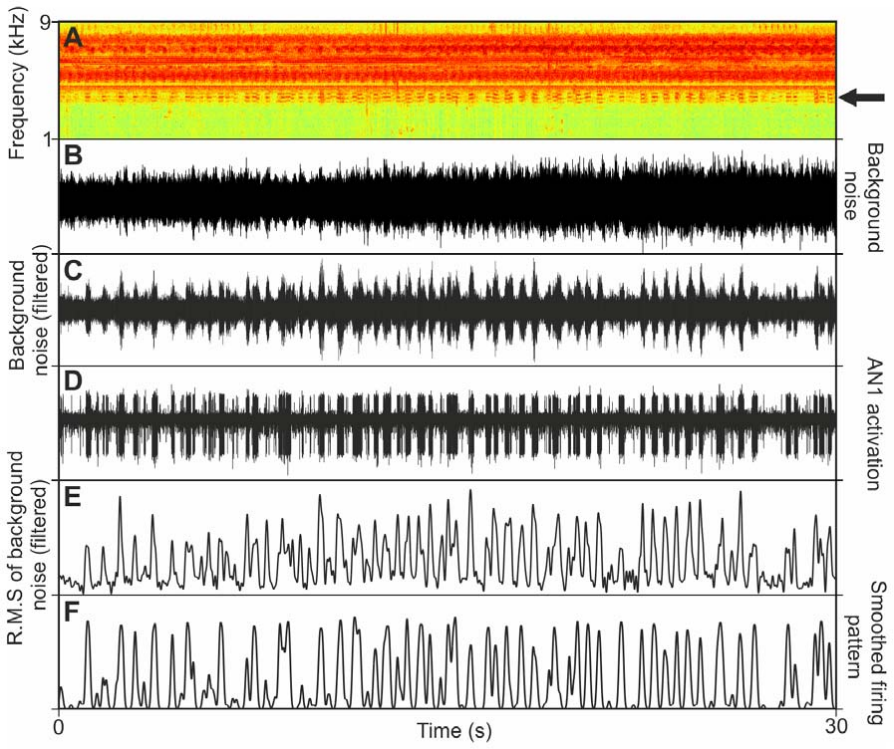
Selective response of AN1 in *Diatrypa* sp. towards conspecific signals embedded in noise outdoors. Sonogram (A) and oscillogram (B) of a 30 seconds section of nocturnal background noise recorded simultaneously with AN1 activity (D) in the natural habitat. (C) Amplitude modulation resulting from filtering the signal in (B) with the species-specific AN1 filter function of *Diatrypa* sp. (standardized tuning curve), revealing calling songs of various males at different distances from the preparation (see arrow in A). Note the high correlation between the RMS amplitude of the filtered noise (E) with the firing pattern of AN1 (F).

Finally, we investigated the quality of neuronal representations of conspecific signals under masking background noise in outdoor recordings of AN1 activity, by correlating this activity with either the complete spectrum of nocturnal noise, or the filtered noise. Fig. 5 shows a representative section of 30 seconds of nocturnal background noise recording as sonogram and oscillogram, respectively (A, B), where the latter shows almost no amplitude modulation with the complete spectrum between 1 and 9 kHz. Filtering of this sound section with a filter function derived from the average standardized AN1 tuning curve of *Diatrypa* sp. (C) reveals an amplitude modulation which coincides quite well with the bursting activity of the AN1 (D), where the majority of AN1 bursts were elicited by sound events occurring only in the small frequency band of approximately 1 kHz between 3.5 and 4.5 kHz, representing calling songs of several Diatrypa males at various distances from the preparation. This match is expressed in a strong correlation coefficient of 0.78 between the RMS amplitude in this frequency band (E) and the smoothed firing pattern of AN1 (F; average correlation coefficient of five preparations 0.73±0.03). By contrast, the correlation between the RMS amplitude of the complete spectrum and AN1 activation revealed a correlation coefficient of only 0.1. Thus, the high incidence of single sound elements in the narrow frequency window with the neuronal firing pattern reflects the excellent performance of the AN1 filter in reducing background noise, especially towards higher frequencies.

### Gain control

**Figure 6:**
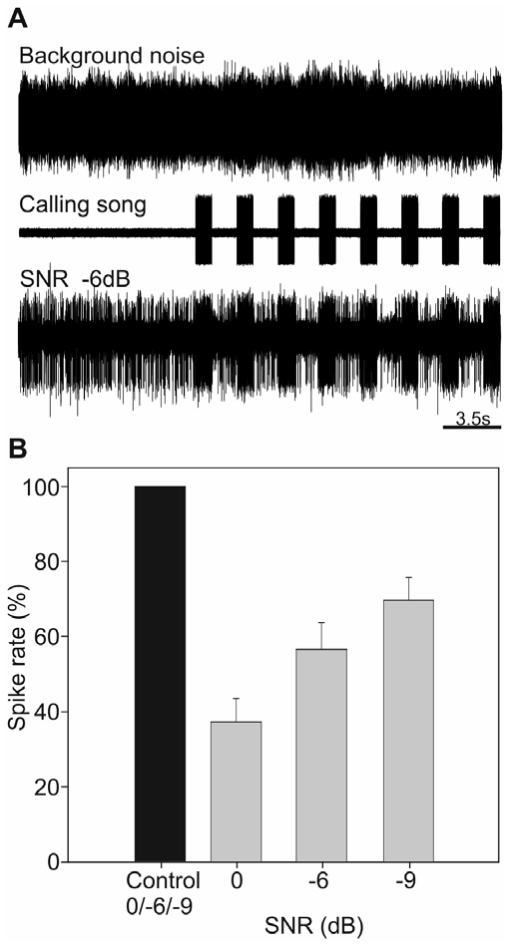
Signal representation in neuronal activity is enhanced by a gain control mechanism. (A) Representative neuronal response of AN1 in *P. podagrosus* to conspecific calling songs under masking noise (SNR −6 dB). Note reduced action potential activity during interstimulus intervals (ISI) compared with the noise-alone situation. (B) Quantification of suppression of the response to noise for three different SNRs (N = 8). Grey bars show the average spike rate during ISI compared with noise-alone (black bar, control). The average stimulus intensity in all experiments was 54.4±1.2 dB SPL.

Our neurophysiological results on the masked ipsi- and contralateral thresholds would suggest that for a receiver the signal representation in background noise is surprisingly reliable at rather low signal-to-noise ratios. Apart from the auditory filter selectivity of the sensory system, and the spatial release from masking a third proximate mechanism might contribute to the excellent neuronal representation of the calling song under background noise, evident in the recording shown in Fig. 6A. When the preparation was stimulated with background noise alone, at a SNR of −6 dB, the noise induced ongoing action potential activity in the AN1 neuron. Each stimulation with the conspecific calling song elicited an even stronger response in the neuron, but remarkably, in the interstimulus intervals the response to the background was considerably reduced compared with the situation before the series of stimuli started. This phenomenon enhanced the contrast between the responses of the cell to the background and the conspecific stimulus and thus may serve as another proximate mechanism for reliable stimulus presentation. The overall effect of the mechanism for various SNRs is summarized in Fig. 6B. Compared with the control situation of continuous background noise the firing rate during the interstimulus intervals decreased significantly on average by 63% at a SNR of 0 dB SPL (paired t-test, t = −4.632, p = 0.002, N = 8). For higher noise levels (−6 and −9 dB, respectively) the degree of suppression of the noise response is reduced, but still amounts to 43% and 30% of the control. At the SNR of −9 dB the spike rate reduction in the interstimulus interval (ISI) of 30% compared with the background noise-only situation seems rather high, considering that the signal detection threshold for *P. podagrosus* achieved only −8.2 dB on average (see Fig. 3, ipsilateral masker position M1/M2). Therefore, we would expect no difference between the ISI and noise-only situation at such high level of background noise. However, for the eight individuals investigated here the masking threshold was on average −11.3 dB and thus the gain control mechanism is still effective.

## Discussion

Singing insects in tropical rainforests are often confronted with call frequency overlap and masking interference due to acoustic competition. Therefore they have to solve cocktail-party-like problems in order to ensure successful communication with conspecifics. In this study we have documented receiver strategies in the auditory pathway of tropical crickets which may counteract the masking effects of background noise. We have identified three mechanisms which contribute to the excellent performance of extracting conspecific signals embedded in acoustic background.

In a previous study we compared the tuning of the homologous AN1 neurons in *P. podagrosus* and two species of European field crickets, where almost no competition for the acoustic communication channel does exist. Indeed, the rainforest species exhibited a more selective tuning compared with the one in its European counterparts [28]. When comparing the filter properties of the AN1 in *Diatrypa* sp. (Fig. 2) with the one in *Paroecanthus podagrosus* we find a strong similarity. In both tropical cricket species the higher selectivity is mainly due to the increased steepness of the slope towards higher frequencies. If the filter has been shaped by natural selection to avoid masking interference, this is exactly what one would expect to happen, because in the cricketś habitat there is more masking potential in the noise spectrum at higher compared with lower frequencies (see spectra in Fig. 1). The quantification of the filter performance, by implementation of the specieśAN1 tuning into audio software and filtering conspecific signals embedded in natural background noise revealed a significantly better performance of the rainforest cricket in representing the important amplitude modulation of the signal [28]. How these rainforest crickets achieve the higher selectivity is currently unclear; in *P. podagrosus* it appears not to result from central nervous shaping of tuning (e.g. through inhibitory side-bands as shown for a katydid; [47]), since the receptor fibers in the ear exhibit the same tuning as the second order AN1 neuron.

Thus, a first step in achieving a high performance of signal detection in high background noise for the two species of cricket is to reduce the amount of acoustic energy that might interfere with that of relevant signals in the communication channel. Therefore the notion that these species suffer from overall noise levels of 55 dB SPL is not correct if we consider these filters. In fact, the RMS-amplitude of the nighttime rainforest noise would be reduced by about 21 dB to values of 34 dB SPL when implementing the AN1 filters in audio software. It illustrates the warning by Brumm and Slabbekoorn [48] that in many studies background noise level measurements are made without considering the critical bandwidth of the signal for a perceptually relevant ratio. In these cases overall SPL measurements of the noise do not tell us very much about the limits of hearing outdoors. In our experiment with masker M3, which includes an additional noise band within the respective filter function of AN1 resulted in a significant decrease of the SNR to about 0 dB. Altogether, our results have shown that the concept of matched filters, using tuning curves and Q-values is rather relevant, and the warning that nervous systems do not perform such frequency analysis [49] is not applicable to the cricket species studied here.

### Spatial release from masking

Like in humans and other vertebrates, our results have further demonstrated that the spatial separation of signal and masker does improve the detection of the signal. When we followed the conventional protocol for such experiments, by using as playback the relevant masker (nocturnal background noise at the time when the insects communicate) first from the same direction as the signal, and then from a different (contralateral) direction, the amount of spatial release from masking was between 6 – 9 dB. These values are within the range of values reported in previous studies and different taxa, using behavioral and neurophysiological approaches [50], [51], [52]. These results were not unexpected since a previous study had shown for *P. podagrosus* that the specieś directional hearing provides large binaural differences, which are strongly frequency dependent and closely matched with the BF of sensitivity at 3.9 kHz [28]. Thus, when the masker is shifted to contralateral, less acoustic energy will be available at the ipsilateral ear for masking, owing to the peripheral directionality. In addition to this peripheral directionality, central nervous processing through lateral inhibition may increase the amount of release from masking, as indicated from values of 2.9 dB for auditory nerve fibers and 9.4 dB for units in the frog torus semicircularis, respectively (a homolog of the inferior colliculus; [53], [52]). Since the AN1 neuron used in our study is a second order interneuron and receives contralateral inhibition in the auditory neuropil of the prothoracic ganglion [54], [55] it is likely that the values for spatial release from masking are due to a combined effect of peripheral directionality and central nervous processing.

Spatial release from masking was not addressed directly in previous studies on insects, but some results indicate that the mechanism is not effective in all taxa (such as grasshoppers; [34]). In contrast, katydids with their known high peripheral directionality and contrast enhancement through lateral inhibition along the longitudinal body axis would provide the proximate basis for spatial release from masking (review in Gerhard and Huber 2002 [11]; Hedwig and Pollack 2008 [56]). In one study in the katydid *Tettigonia viridissima* the representation of up to three acoustic signals was investigated in the responses of a pair of local interneurons (omega cells), while varying the direction of these signals [3]. The results suggest that the auditory world of the katydid is rather sharply divided into two azimuthal hemispheres, with signals arriving from any direction within one hemisphere being predominantly represented in the discharge of neurons of this side of the auditory pathway. Future experiments with a signal and masker thus are expected to reveal even higher values for spatial release from masking in katydids compared with crickets, due to stronger inhibitory interactions.

### Outdoor experiments

Our results with the two species of tropical rainforest cricket have demonstrated a remarkable low SNR at the masked threshold in the natural habitat, where they have to listen to conspecific signals at a mean background noise level of 55 dB SPL. SNR-values were rather similar with about −22 dB and −23 dB for *P. podagrosus* and *Diatrypa* sp., respectively. Given that the absolute sensitivity (as measured in the undisturbed lab situation) of both species is approximately 33 dB SPL at the BF of 3.9 kHz, these low SNRs mean that the threshold for detecting the conspecific signal is almost unaffected by the background noise in real world situations. The high correlation values of filtered background noise with the neuronal representations in receivers of *Diatrypa* sp. revealed that only a small portion of acoustic energy in the habitat elicited an AN1 response, owing to the increased frequency selectivity (see Fig. 5). However, it should be noted that both cricket species investigated here were also favored in the detection of conspecific calling songs due to the low acoustic energy within the frequency channel around the receivers BF which seems to be a rather typical spectral feature of the rainforest on BCI.

A major and unexpected finding in our study was the difference in SNRs obtained under natural conditions (values of about −23 dB) compared with those in the lab were SNRs yielded on average only −14.5 and −16 dB in both species. Background noise level measurements in the lab and outdoors were performed in the same way; in both situations the microphone was placed at the position of the ipsilateral ear and revealed an average noise level of 55 dB SPL. However, in the laboratory experiments the ipsilateral ear was facing directly towards a single speaker broadcasting a highly complex auditory scene of nocturnal background noise. Such single speaker playbacks apparently do not properly reconstruct the noise situation in a spatially realistic manner while in the natural habitat its multiple sound sources are spatially distributed in space (see review of acoustic playback techniques by Douglas and Mennill [57]).

Thus, under natural conditions where the masking noise acts on the receiver from all directions, the SNR in the masked condition is almost identical to the unmasked threshold in the lab ([28] and Fig. 2). As a consequence, and in addition to the reduction of the masking problem due to selective filtering the problem is further reduced by the spatial separation of all relevant noise sources. Our findings are fully consistent with the warning by Bee & Micheyl [12] that “an approach using one or a limited number of masking noise sources in highly controlled laboratory studies of spatial unmasking does not wholly reflect the real-world listening conditions that many animals face”.

### Gain control

The third mechanism that contributes to the high performance of signal detection in the tropical crickets is based on a specific membrane property of nerve cells, and provides a gain-control for representing only one of several alternative signals in the nervous response. For crickets [36], [58] and katydids [3] such a neuronal mechanism has been described to be particularly effective in receiver situations, where more than one signaler, or a conspecific signaler and noise sources, are broadcast from one auditory side, not unusual in populations of crickets and katydids [59], [60], [61]. The underlying synaptic mechanism is a hyperpolarisation with a slow build-up and decay time [36] and involves a calcium-activated potassium current [62], [63]. Since the inhibition prevents suprathreshold depolarization of the membrane in response to softer signals or background noise, it represents a gain control effectively filtering out irrelevant or competing signals. A quantification of the effect in reducing activity to the background revealed suppression by 30 – 60%, depending on the SNR (Fig. 6). In this way, the gain control enhances the contrast between the response to the background and signal in a situation where spatial release from masking is not effective, because both act from the same direction.

A final problem could only occur if species such as *P. podagrosus* and *Diatrypa* sp., with an almost identical CF of the calling song at 3.8 kHz and 4 kHz, respectively and the same BF of sensitivity at 3.9 kHz would communicate at the same time and location. As shown in two studies on katydids, in a situation when only two species use a spectrally similar signal, this can result in complete suppression of calling activity of one species by the other, or a shift in the diurnal calling activity of one species [64], [65]. However, in an extensive survey of cricket calling songs at different locations, different times of the night and heights within the rainforest we never experienced such a situation: when *Diatrypa* sp. was calling in the background, as in the recording shown in Fig. 5, we never recorded calling songs of *P. podagrosus*, and *vice versa* (Schmidt et al. in preparation). Thus, acoustic niche partitioning in time and space serves as an additional mechanism at the sender side to reduce acoustic interference of signals of similar CF [14], [15], [66], [67].

In summary, despite the original assumption that the situation in a nocturnal tropical rainforest looks terribly complicated for any involved taxon to communicate acoustically, due to masking interference, our data have shown that a combination of three mechanisms in the receiver, namely selective frequency filtering, spatial release from masking and a gain control mechanism, all contribute to improve the neuronal representation of conspecific signals in receivers.
